# Mastectomy rates are decreasing in the era of service screening: a population-based study in Italy (1997–2001)

**DOI:** 10.1038/sj.bjc.6603405

**Published:** 2006-10-17

**Authors:** M Zorzi, D Puliti, M Vettorazzi, V De Lisi, F Falcini, M Federico, S Ferretti, I F Moffa, L Mangone, M P Mano, C Naldoni, A Ponti, A Traina, R Tumino, E Paci

**Affiliations:** 1 Istituto Oncologico Veneto, Padova, Italy; 2 Clinical and Descriptive Epidemiology Unit-CSPO-Research Institute of the Tuscany Region, Firenze, Italy; 3 Parma Cancer Registry, Parma, Italy; 4 Romagna Cancer Registry, Forlì, Italy; 5 Modena Cancer Registry, Modena, Italy; 6 Ferrara Cancer Registry, Ferrara, Italy; 7 Epidemiology Unit-ASL 2, Perugia, Italy; 8 Reggio-Emilia Cancer Registry, Reggio-Emilia, Italy; 9 University of Turin-Department of Biological Sciences and Human Oncology, Turin, Italy; 10 Screening program-Emilia-Romagna Region Health Department, Bologna, Italy; 11 Epidemiology Unit-CPO Piemonte, Turin, Italy; 12 Department of Oncology-ARNAS Ascoli, Palermo, Italy; 13 Cancer Registry and Human Pathology Department-Arezzo Hospital, Ragusa, Italy

**Keywords:** breast cancer screening, breast conserving surgery, screening mammography

## Abstract

We enrolled all 2162 *in situ* and 21 148 invasive cases of breast cancer in 17 areas of Italy, diagnosed in 1997–2001. Rates of early cancer increased by 13.7% in the screening age group (50–69 years), and breast conserving surgery by 24.6%. Advanced cancer rates decreased by 19.4%, and mastectomy rates by 24.2%. Service screening did not increase mastectomy rates in the study population.

In 2001, the authors of the Cochrane review of mammographic screening published on the Lancet website claimed that screening is associated with an increase of mastectomy rates (Olsen *et al*, 2001). In 2002, a study of changes in surgical treatment of breast cancer in the city of Florence (Paci *et al*, 2002) concluded that breast conserving surgery (BCS) was beneficial and showed a decreasing trend in mastectomy rates. In a subsequent paper considering the harms and benefits of screening, Gotzsche (2004) asserted that there would be about 20% more mastectomies when women are screened than if they are not screened.

The purpose of this paper is to evaluate the changes introduced by service screening in the use of BCS and mastectomies in the period 1997–2001. We focused on the appropriateness of the surgical approach to cases where BCS was recommended in accordance with the existing guidelines (FONCaM, 2001). We present the trends of the mastectomy and BCS rates in Italian areas covered by a population-based registry, with or without a service-screening programme.

## MATERIALS AND METHODS

The study included all breast cancers diagnosed between 1997 and 2001 in women aged 40–79 years who were resident in 17 areas mainly located in central and northern Italy. The characteristics of both the breast cancer screening programmes and main performance indicators have been described in detail (Giordano *et al*, 1996).

In all areas, a registry was active at the start of screening. Cases were included according to the IARC rules for cancer registration (Zanetti *et al*, 2002). *In situ* carcinomas were included, whereas death certificate only and multiple primaries were excluded.

All breast cancers were classified by size and nodal status according to the TNM-UICC classification and on the basis of the data reported by each local centre (Sobin and Wittekind, 1997).

Breast cancer cases, *in situ* or invasive, with a size of 30 mm or less were classified as ‘early’. Invasive cancer cases with sizes greater than 30 mm, irrespective of nodal status, were classified as ‘advanced’. Surgical treatment was classified in two categories: breast conserving surgery (including excisional biopsy, wide local excision and quadrantectomy) and mastectomy (including all types of mastectomy).

All registry-based breast cancer cases were linked to the screening file and divided by detection method. We divided cases primarily as either screen-detected (SD) or not screen-detected (NSD). Cases in the NSD division were further divided so that there were four main case divisions. They were:

SD cases

1. Having a tumour detected in the first or subsequent round at the first screening test and cases having a tumour detected at a repeated screening test. NSD cases
2. Diagnosed clinically outside the screening process following a negative screening test (includes interval cancer cases).
3. Diagnosed within women who never responded to their invitation – the never-respondent case division.
4. Diagnosed before an invitation could be sent (as it took several years to achieve full coverage of the population with an invitation to screening) – the not-yet-invited case division.

We performed an intention-to-treat, non-randomised analysis comparing the not-yet-invited case division with the combination of divisions 1, 2 and 3 – all invited women.

Incidence rates of cancer and surgical treatments were calculated using data on populations by study centre and year produced by the National Statistics Institute.

The association between independent variables and surgery was assessed by means of multivariate logistic regression, using the STATA 7.0 (Stata Corp, 2001) statistical package with a *P*<0.05 considered statistically significant.

## RESULTS

Seventeen areas from six different regions took part in the study. In total, we enrolled 2162 *in situ* and 21 148 invasive breast cancer cases incident in the period 1997–2001 (Table 1).

Overall, 61.1% of cases underwent BCS, with an increase during the study period from 53.8% in 1997 to 65.6% in 2001 (Table 2). Women aged 70–79 years showed the lowest proportion of BCS (44.1%) and also the largest proportion of cases without any surgical therapy (5.9%), whereas no relevant differences were observed between the younger age classes.

The proportion of BCS was highest for the *in situ* and invasive breast cancer cases ⩽10 mm, and progressively decreased starting from cases pT1c (71.5%), with a drop between cases with pT2 ⩽ and >30 mm (46.2 and 23.3%, respectively). Only 58.4% pT1micr received BCS.

Breast conserving surgery was carried out in more than 75% of SD cases at first test and in 83% of those SD at subsequent tests, compared to 54.4 and 52.5% in cases not detected by screening – case divisions not-yet-invited and never-respondent, respectively.

The multivariate logistic odds ratio (OR) of receiving a mastectomy *vs* a BCS decreased by 10% per year between 1997 and 2001, and increased progressively with age: in women aged 70–79 years, it was more than double than in women aged 40–49 years (Table 3).

As compared to not-yet-invited cases, the probability of mastectomy was reduced by more than one-third in SD cases at first test (OR 0.65, 95% CI 0.58–0.72) and almost halved in those SD at subsequent tests (OR 0.53, 0.45–0.62), whereas participation in screening in the past reduced the risk of mastectomy by 12% (*P*=0.09). The OR for mastectomy was significantly lower for women who were invited to screening than for those who had not yet been invited (OR 0.77, 95% CI 0.71–0.85), using an intention-to-treat analysis.

**Figure 1:**
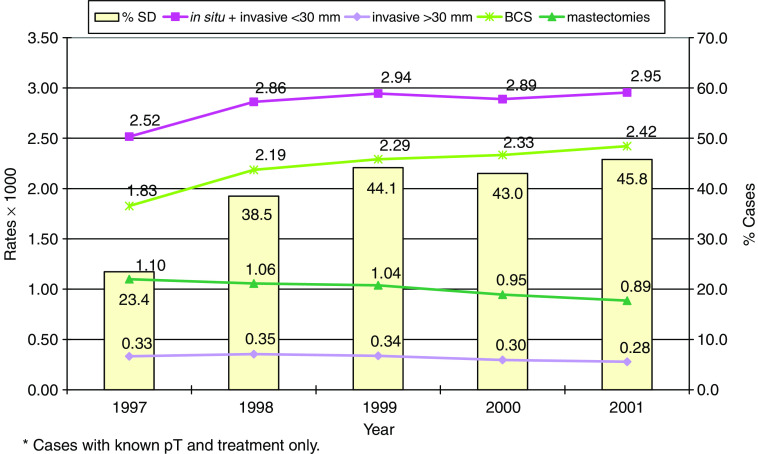
Women aged 50–69 years: trends of incidence rates (per 1000) of breast cancer cases and surgical interventions^*^. Proportion of SD cases (first and subsequent) by year.

Figure 1 shows the incidence rate trends per 1000 women of BCS and mastectomies in those aged 50–69 years, the target of service screening. During the observation period, the proportion of SD cases (prevalence and incidence tests) in women aged 50–69 years old increased from 23.4 to 45.8% of the whole incidence rate. Rates of early breast cancer cases increased by 14.9% as compared to an increase by 24.6% of BCS rate, whereas the incidence of advanced cancers decreased by one-fifth (19.4%) with a 24.2% decrease of mastectomy rates.

The reduction of mastectomy rates was due to a decreased proportion of mastectomies in early cases (from 31 to 21%, *P*<0.001) combined with an increase from 73 to 76% in those advanced (*P*=0.95). Also in the 40–49 years age group, the proportion of early cancer cases and BCS increased by 13.1 and 20.9%, respectively.

## DISCUSSION

In this study, we analysed the impact of the spread of screening programmes on breast cancer incidence rates and surgical treatment modality on the whole population in the period 1997–2001. In the 50–69 years group, the proportion of SD cases increased.

In all age groups, we observed an increase in incidence rates of *in situ* and invasive tumours ⩽30 mm and reduction or stability of larger invasive cases; both these variations were much larger in women aged 50–69 years invited to participate in service screening. Overall, the proportion of cases which received BCS increased by 12% (from 54 to 66%).

The increase of BCS is the combined effect of the increase in the rates of early tumours, owing to the expected excess of early breast cancer detected after the invitation to screening, and the improving appropriateness of BCS. Rates of BCS and early tumours went in parallel and the appropriateness of surgical treatment with BCS was especially evident in SD cases and cases NSD but with a previous screening test. There was also a clear relationship between the decreasing trend of advanced invasive tumours in the population and the decreasing rates of mastectomies.

In all age classes, BCS rates increased by more than 20% and mastectomy rates decreased by even higher proportions. However, in women aged 50–69 years, we observed a 24% reduction in mastectomy rates, notwithstanding the increasing proportion of mastectomies appropriately carried out in large invasive cases (from 73 to 76%).

The proportion of cases receiving a mastectomy was directly associated with increasing tumour size, except for cases with a micro-invasive component, who underwent mastectomy in more than 40% of cases, probably because of the *in situ* multifocal component which was responsible for the indication of mastectomy.

In the Netherlands, no increase was observed in the use of BCS for patients 50–69 years of age across a 9-year period (64% both in 1990 and 1998), but proportions were used and not population incidence rates as in this paper (Ernst *et al*, 2001). In a recent study in Australia (Samnakay *et al*, 2005), of all patients undergoing surgery for breast cancer within service screening, the 59.5% in the screen group and 42.3% in the non-screen group had BCS.

Results from this large population-based study in Italy reject the hypothesis that service screening increases mastectomy rates in the population. On the contrary, our data confirm that the introduction of service screening brought about a reduction in mastectomy rates and improved the appropriateness of treatment of early tumours with BCS.

## Figures and Tables

**Table 1:** Study centres and breast cancer cases included, by regional area

			**Cases (no.)**		
**Region**	**Centre**	**Screening activation**	***In situ***	**Invasive**	**Total**
Emilia Romagna	Bologna City	June 1997	141	1819	1960
	Bologna North	November 1997	97	665	762
	Cesena	December 1997	63	605	668
	Ferrara	October 1997	109	1462	1571
	Forlì	March 1996	91	635	726
	Modena	October 1995	319	2281	2600
	Parma	July 1997	199	1514	1713
	Ravenna	December 1995	177	1401	1578
	Reggio Emilia	November 1994	184	1557	1741
	Rimini	November 1997	60	883	943
Piemonte	Torino	February 1992	170	1642	1812
Sicilia	Palermo	—	56	1443	1499
	Ragusa	February 1994	15	582	597
Toscana	Florence City	October 1990	109	1467	1576
	Florence suburbs	May 1992	56	641	697
Umbria	Perugia	November 1997	87	1041	1128
Veneto	Verona	July 1999	229	1510	1739
					
Total			2162	21 148	23 310

**Table 2:** Distribution of breast cancer surgical treatment by year of diagnosis and method of detection

			**Surgical treatment (row %)**			
	**Number of cases (%)**		**Conservative**	**Mastectomy**	**Not performed**	**Not reported**
*Year of diagnosis*						
1997	4031	17.3	53.8	39.6	3.5	3.1
1998	4402	18.9	58.7	36.3	3.1	1.9
1999	5130	22.0	62.1	33.5	2.5	1.9
2000	5074	21.8	64.0	31.9	2.7	1.5
2001	4673	20.0	65.6	30.1	2.6	1.7
						
Total	23 310	100.0	61.1	34.1	2.8	2.0
						
*Method of detection*						
All invited	9953	42.7	69.9	26.9	1.8	1.4
SD at first test	3910	16.8	75.7	23.1	0.5	0.7
SD at subsequent tests	1987	8.5	83.0	16.0	0.5	0.6
NSD with a previous test	1647	7.1	65.9	31.1	1.8	1.2
NSD never-respondent	2409	10.3	52.5	39.0	5.2	3.3
Not-yet-invited	13 357	57.3	54.5	39.4	3.6	2.5

NSD=not screen-detected; SD=screen-detected.

**Table 3:** Multivariate logistic analysis of the probability of receiving a mastectomy by year of diagnosis, age, tumour size and detection method^a^

	**OR^b^**	**95% CI**	***P*-value**
*Year of diagnosis* c			
Common OR for a unit increase	0.90	0.88–0.93	<0.001
			
*Age (years)*			
40–49^d^	1.00	—	—
50–59	1.15	1.02–1.29	0.018
60–69	1.31	1.16–1.47	<0.001
70–79	2.15	1.93–2.39	<0.001
			
*pT*			
PTis	0.60	0.53–0.68	<0.001
pT1micr	1.90	1.54–2.35	<0.001
pT1a	0.76	0.64–0.91	0.002
pT1b	0.52	0.47–0.58	<0.001
pT1c^d^	1.00	—	—
pT1NOS	0.95	0.66–1.38	0.79
pT2 ⩽30 mm	2.83	2.61–3.09	<0.001
			
*Method of detection*			
NSD not yet invited^d^	1.00	—	—
SD at first test	0.65	0.58–0.72	<0.001
SD at subsequent tests	0.53	0.45–0.62	<0.001
NSD with a previous test	0.88	0.76–1.02	0.09
NSD never respondend	1.19	1.05–1.35	0.007
			
*Log likelihood*	−10 176.3, P<0.0001		

CI=confidence interval; NSD=not screen-detected; OR=odds ratio; SD=screen-detected.

OR and 95% CI.

a Excluded cases with unknown treatment or not operated and cases with pTX or pTunknown or pT>30 mm.

b Adjusted by multivariate regression for each of the variables in the table.

c Linear assumption.

d Reference.
